# Supplementary material for: Underestimated barrier effects of ocean fronts shape global fishery distribution
Source: Nat Commun. 2026 Mar 28;17:4545. doi: 10.1038/s41467-026-71250-0 (PMC13194931; doi:10.1038/s41467-026-71250-0)
Supplement: Supplementary file 1 — Supplementary Information [file 41467_2026_71250_MOESM1_ESM.pdf]

Supplementary Information for

Underestimated barrier effects of ocean fronts shape global fishery distribution

Qinwang Xing[a,b,c,d,e*], Zihui Gao[a,b], Shin-ichi Ito[b], Haiqing Yu[f], Bilin Liu[a,c,d,e], Heng Zhang[g], Xinjun Chen[a,c,d,e*], Wei Yu[a,c,d,e*]

*Corresponding author. Email: qwxing@shou.edu.cn; xjchen@shou.edu.cn; wyu@shou.edu.cn

This file includes:

Supplementary Figs. S1 to S7

Supplementary Table S1 to S2

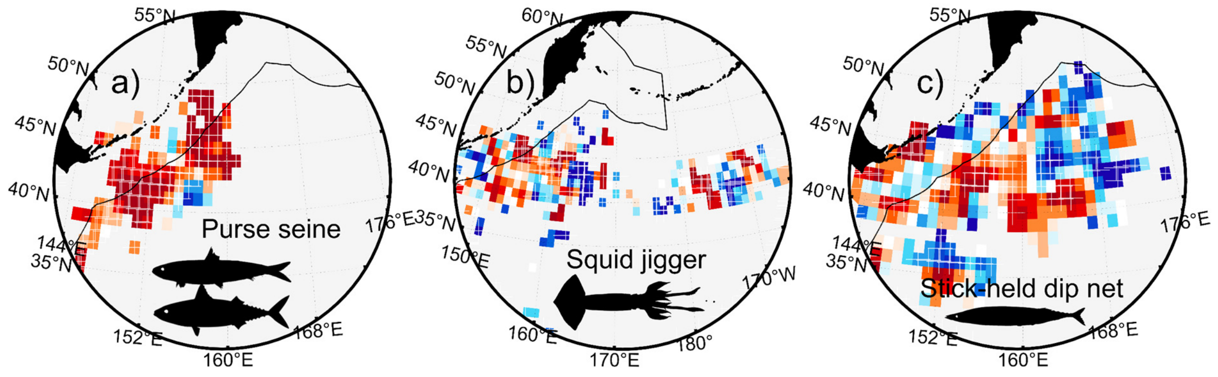

Fig. S1 Spatial distribution of relative difference in fishing effort per unit area (RD_{FPA}) between frontal and non-frontal zones for the four species, calculated from deep learning–derived fishing effort. White plus signs indicate areas with statistically significant RD_{FPA}, and grey areas denote regions with no data. Red areas indicate higher fishing effort in frontal zones relative to non-frontal zones, while blue areas indicate the opposite. Source data are provided as a Source Data file.

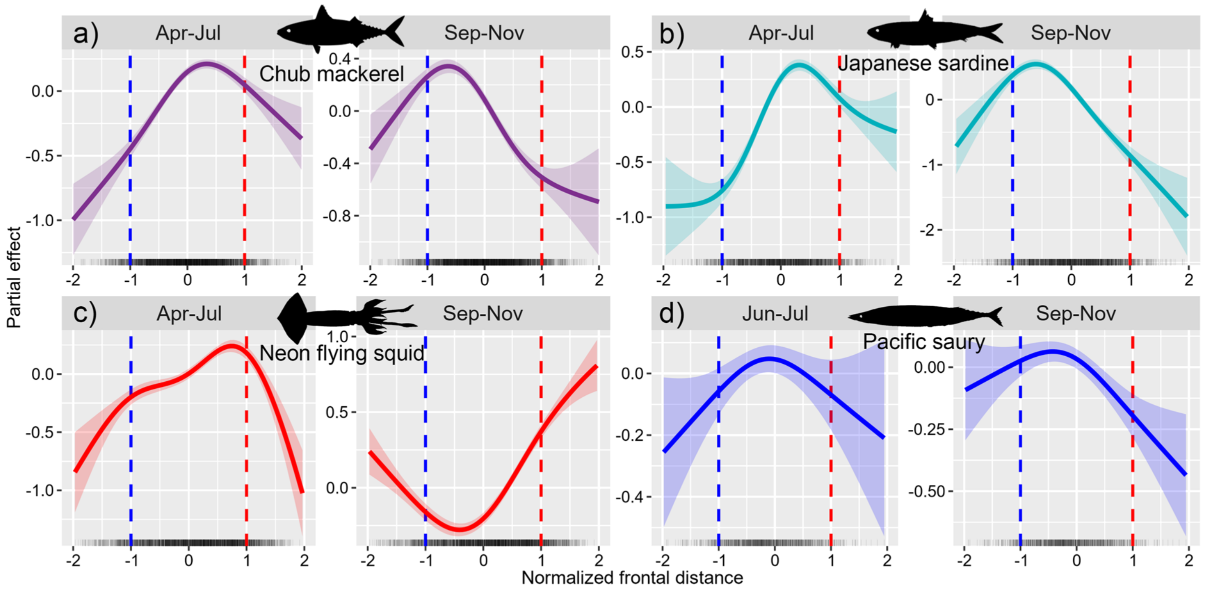

Fig. S2 Partial effects of generalized additive model smooth terms for normalized distance from fronts before and after August. Curves represent the partial effects for the four species, with shaded areas indicating standard errors. Red and blue dotted lines mark the normalized boundaries of the warm and cold zones, respectively. Source data are provided as a Source Data file.

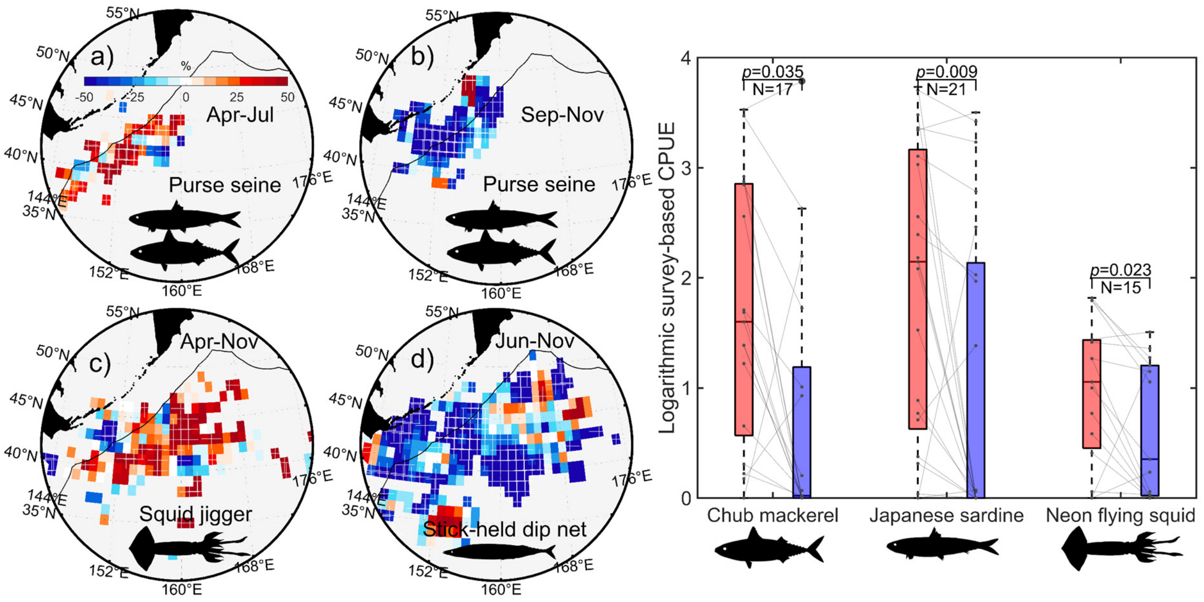

Fig. S3 **a–d**) Similar to Fig. S1, but showing relative difference in fishing effort per unit area (RD_{FPA}) between frontal warm and cold zones. Red areas indicate higher fishing effort in warm zones relative to cold zones, while blue areas indicate the opposite. White plus signs indicate areas with statistically significant RD_{FPA}, and grey areas denote regions with no data. **e**) Comparison of fishery-independent catch per unit effort (CPUE) between frontal warm and cold zones. Lines link paired CPUE values from adjacent warm and cold zones for each species. For chub mackerel and Japanese sardine, CPUE is expressed as log-transformed kg/km^3, while for neon flying squid it is measured in kg/h. Source data are provided as a Source Data file.

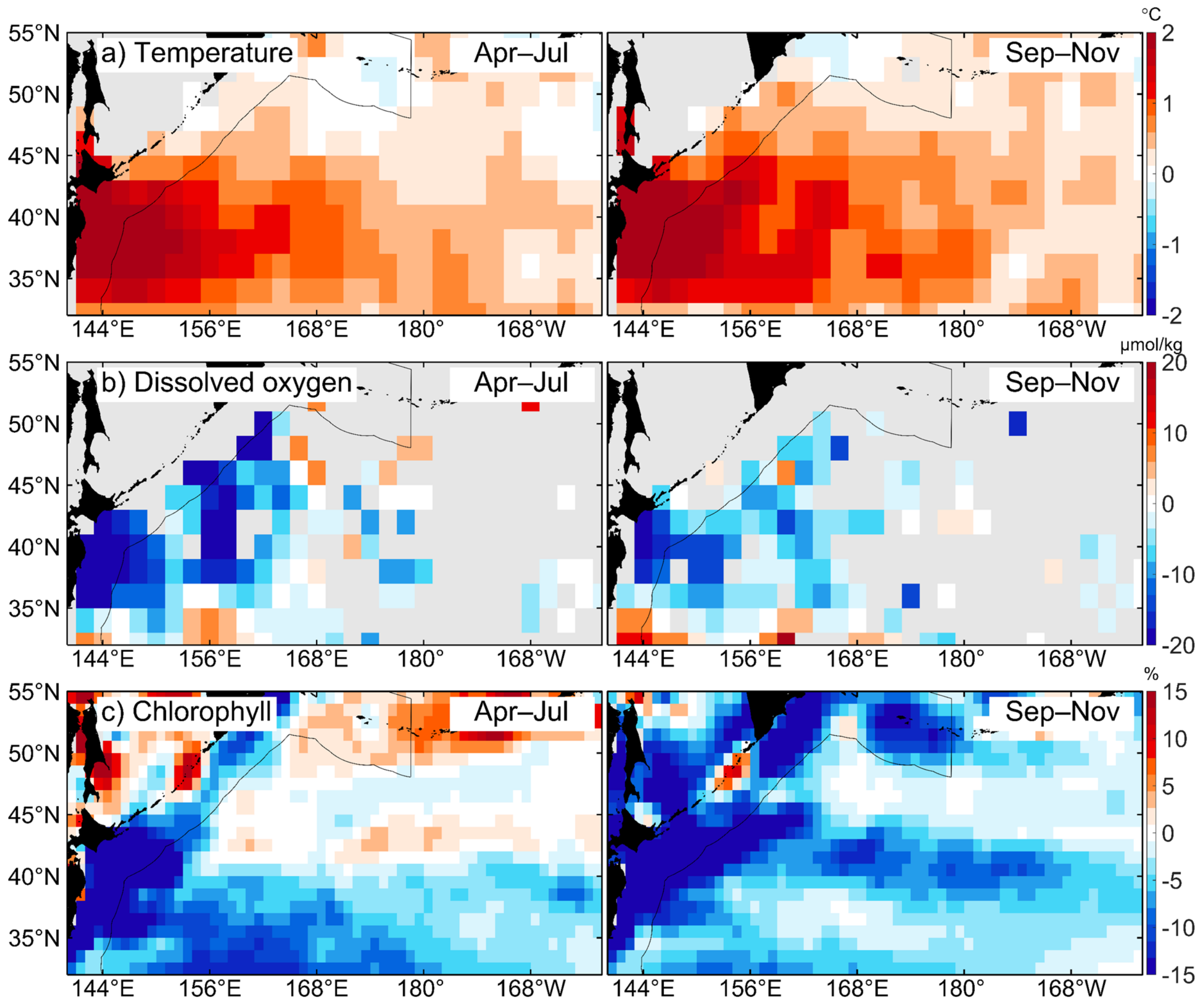

Fig. S4 Environmental differences between frontal warm and cold zones before and after August. Red areas in **a**–**c**) indicate higher pelagic (<200 m) water temperature, dissolved oxygen, and surface chlorophyll-a concentration in warm zones compared to cold zones; blue areas indicate the opposite. Source data are provided as a Source Data file.

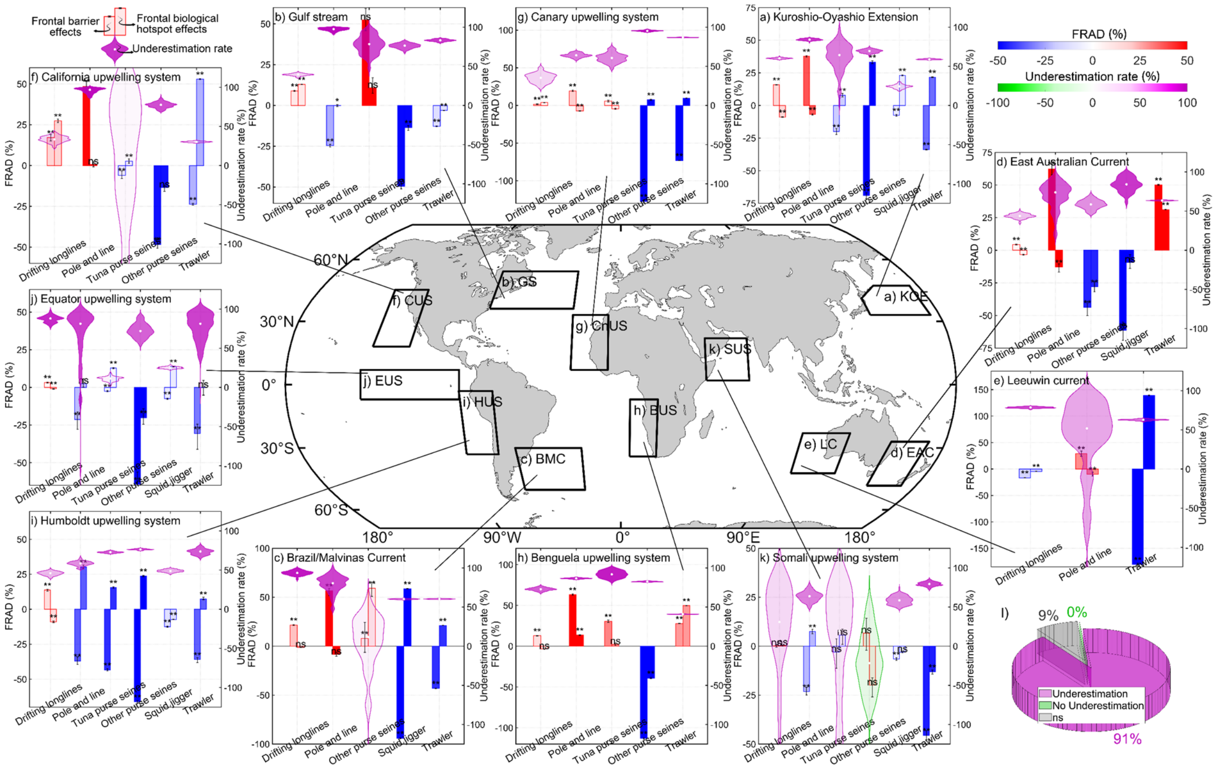

Fig. S5 Frontal biological hotspot and barrier effects on fisheries across different regions. **a**–**k**) Frontal hotspot (left bars) and barrier (right bars) effects across 11 regions and 6 fishing gears, based on the fishing effort relative anomaly difference (FRAD). Positive values indicate higher fishing efforts in frontal versus non-frontal zones (hotspot effects), and in warm versus cold zones (barrier effects). Error bars represent the 25th and 75th percentiles with the central value indicating the median of 1000 simulations, and asterisks denote significance levels (*p < 0.05, **p < 0.001, ns > 0.05). All exact statistics are available in the Source Data. Violin plots show the underestimation rate of frontal fishery effects when barrier effects are ignored, with white dots representing the median. **l**) Percentage of significantly (p < 0.05) underestimated and non-underestimated cases. Source data are provided as a Source Data file.

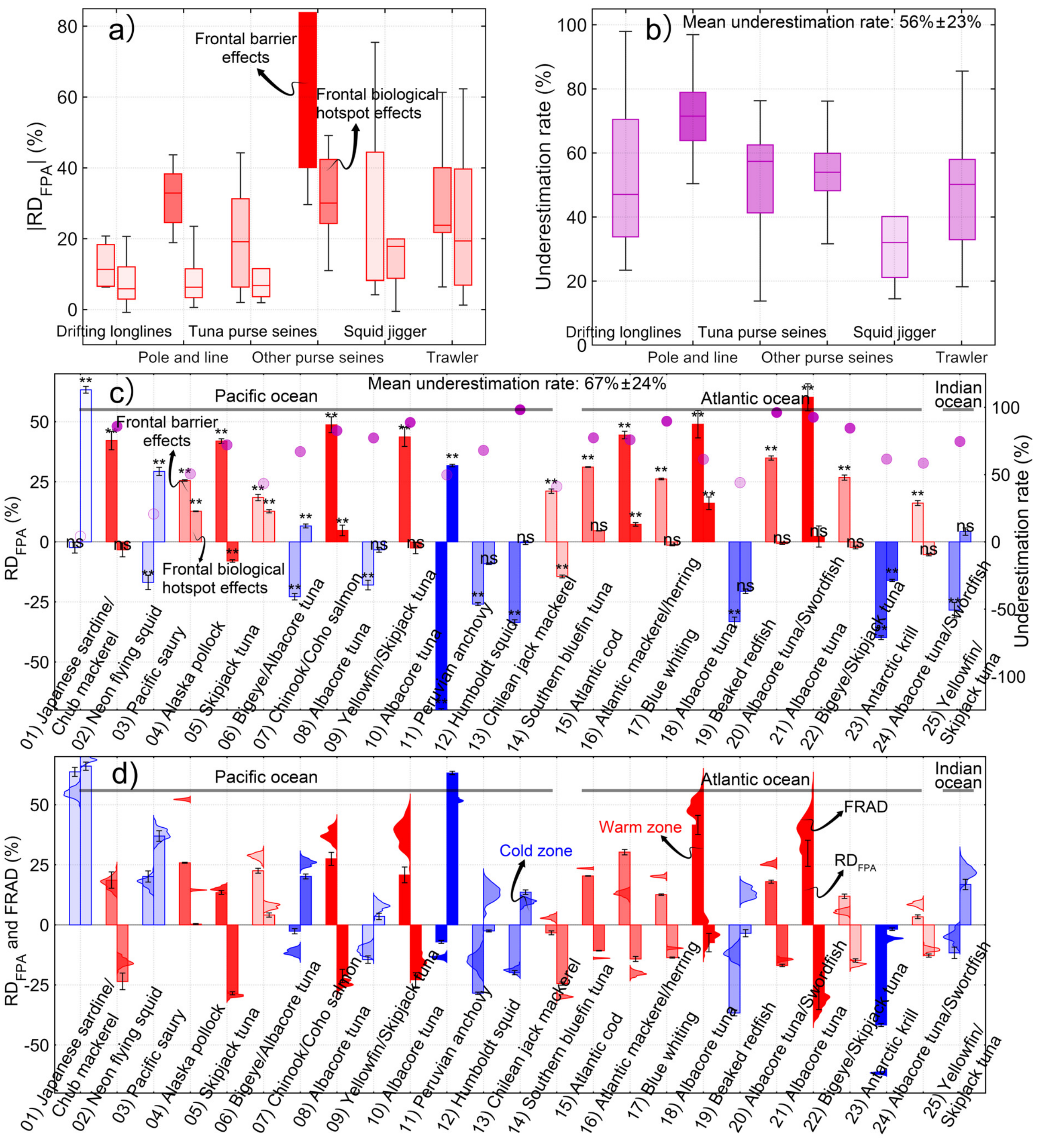

Fig. S6 Frontal biological hotspot and barrier effects on fisheries across different regions and stocks, based on the relative difference in fishing effort per unit area (RD_{FPA}). **a**) Absolute RD_{FPA} between frontal and non-frontal zones (left, hotspot effects) and between warm and cold zones (right, barrier effects) across fishing gears. Boxplots show the non-outlier minimum, 25th, 50th, 75th percentiles, and non-outlier maximum. **b**) Underestimation rate of frontal fishery effects when barrier effects are ignored. **c**) Frontal hotspot (left bars) and barrier (right bars) effects across 25 stocks. Positive values indicate higher fishing efforts in frontal versus non-frontal zones (hotspot

effects), and in warm versus cold zones (barrier effects). Error bars represent the standard deviation with the central value indicating the mean of 1,000 simulations, and asterisks denote significance levels (*p < 0.05, **p < 0.001, ns > 0.05). All exact statistics are available in the Source Data. Purple dots show the underestimation rate of frontal fishery effects when barrier effects are ignored. **d**) Fishery differences between frontal warm (left) or cold (right) zones and non-frontal zones. Bell-shaped areas and bar graphs show the relative difference in fishing effort relative anomaly difference (FRAD) and RDFPA, respectively. Error bars represent the standard deviation with the central value indicating the mean of 1,000 simulations, and asterisks denote significance levels (*p < 0.05, **p < 0.001, ns > 0.05). Source data are provided as a Source Data file.

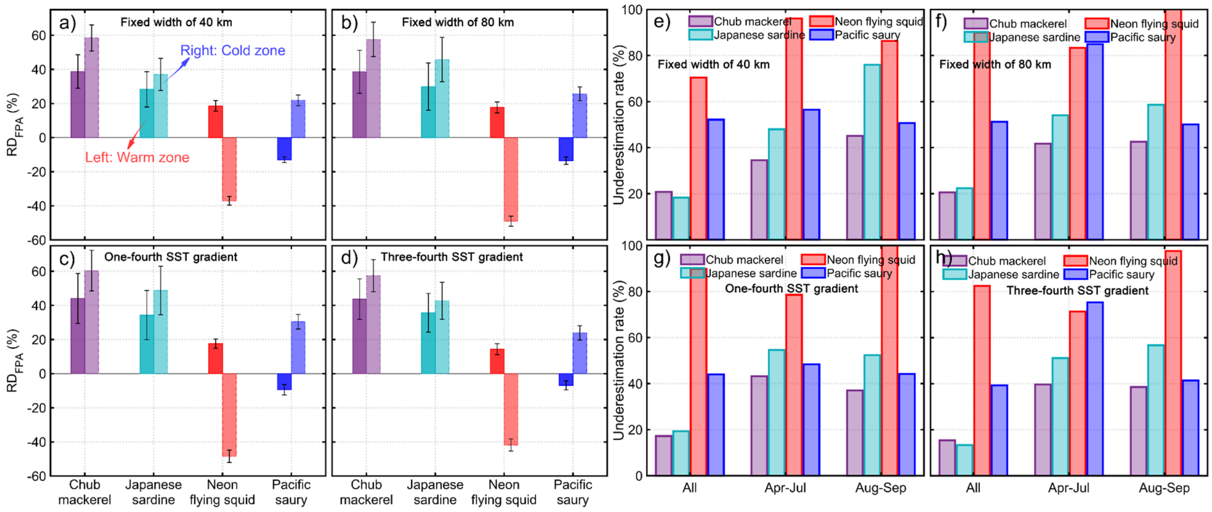

Fig. S7 Sensitivity analyses of the threshold values used for frontal zone detection. **a–d**) Catch differences between frontal warm (left) or cold (right) zones and non-frontal zones using relative difference in fishing catch per unit area (RDFPA). Error bars show standard deviation, and central values denote the mean of 1,000 simulations. **e–h**) Underestimation rate of front-induced variations in fishery distribution across fishing seasons when barrier effects are ignored, calculated as the percentage difference between the absolute values of hotspot and barrier effects relative to the larger of the two using RDFPA. The results in subfigures (**a, e**), (**b, f**) are calculated using frontal zones with fixed widths of 40 km and 80 km, respectively, whereas those in subfigures

(**c, g**), (**d, h**) are derived from frontal zones comprising all pixels where the logarithmic sea surface temperature (SST) gradient magnitude exceeds one-fourth and three-fourths of that at the nearest frontal pixel, respectively. Source data are provided as a Source Data file.

Table S1 Summary of common and scientific names of species mentioned in this study.

Common names	Latin Name
Alaska pollock	*Gadus chalcogrammus*
Albacore tuna	*Thunnus alalunga*
Antarctic krill	*Euphausia superba*
Atlantic cod	*Gadus morhua*
Atlantic herring	*Clupea harengus*
Atlantic mackerel	*Scomber scombrus*
Atlantic salmon	*Salmo salar*
Beaked redfish	*Sebastes mentella*
Bigeye tuna	*Thunnus obesus*
Blue shark	*Prionace glauca*
Blue whiting	*Micromesistius poutassou*
Chilean jack mackerel	*Trachurus murphyi*
Chinook salmon	*Oncorhynchus tshawytscha*
Chub mackerel	*Scomber japonicus*
Coho salmon	*Oncorhynchus kisutch*
Hammerhead shark	*Sphyrna spp.*
Humboldt squid	*Dosidicus gigas*
Pacific jack mackerel	*Trachurus symmetricus*
Japanese sardine	*Sardinops melanostictus*
Neon flying squid	*Ommastrephes bartramii*
Pacific saury	*Cololabis saira*
Peruvian anchovy	*Engraulis ringens*
Pink shrimp	*Farfantepenaeus duorarum*
Rockfish	*Sebastes spp.*

Skipjack tuna	*Katsuwonus pelamis*
Southern bluefin tuna	*Thunnus maccoyii*
Swordfish	*Xiphias gladius*
Yellowfin tuna	*Thunnus albacares*

Table S2 Summary of fisheries targeting 25 key stocks and their gear types. Identification of fishing activities for stocks 1–3 and 12 was based on cross-referencing MMSI records with the North Pacific Fisheries Commission (NPFC) and the South Pacific Regional Fisheries Management Organisation (SPRFMO) registries, respectively.

Stock name	Fishing gear
(1) Japanese sardine/Chub mackerel	Linking MMSI to NPFC records
(2) Neon flying squid	Linking MMSI to NPFC records
(3) Pacific saury	Linking MMSI to NPFC records
(4) Alaska pollock	Trawlers
(5) Skipjack tuna	Pole and line
(6) Bigeye/Skipjack tuna	Drifting longlines
(7) Chinook/Coho salmon	Trollers
(8) Albacore tuna	Trollers
(9) Yellowfin/Skipjack tuna	Tuna purse seines
(10) Albacore tuna	Trollers
(11) Peruvian anchovy	Other purse seines
(12) Humboldt squid	Linking MMSI to SPRFMO records
(13) Chilean jack mackerel	Other purse seines
(14) Southern bluefin tuna	Drifting longlines
(15) Atlantic cod	Trawlers
(16) Atlantic mackerel/herring	Other purse seines
(17) Blue whiting	Trawlers
(18) Albacore tuna	Trollers
(19) Beaked redfish	Trawlers
(20) Albacore tuna/Swordfish	Drifting longlines
(21) Albacore tuna	Trollers

(22) Bigeye/Skipjack tuna	Pole and line
(23) Antarctic krill	Trawlers
(24) Albacore tuna/Swordfish	Drifting longlines
(25) Yellowfin/Skipjack tuna	Pole and line
