# Supplementary material for: Underestimated barrier effects of ocean fronts shape global fishery distribution
Source: Nat Commun. 2026 Mar 28;17:4545. doi: 10.1038/s41467-026-71250-0 (PMC13194931; doi:10.1038/s41467-026-71250-0)
Supplement: Supplementary file 2 — Reporting Summary [file 41467_2026_71250_MOESM2_ESM.pdf]

nature portfolio

Corresponding author(s): Qinwang Xing

Last updated by author(s): Mar 1, 2026

Reporting Summary

Nature Portfolio wishes to improve the reproducibility of the work that we publish. This form provides structure for consistency and transparency in reporting. For further information on Nature Portfolio policies, see our Editorial Policies and the Editorial Policy Checklist.

Statistics

For all statistical analyses, confirm that the following items are present in the figure legend, table legend, main text, or Methods section.

n/a	Confirmed	
☐	☒	The exact sample size (*n*) for each experimental group/condition, given as a discrete number and unit of measurement
☐	☒	A statement on whether measurements were taken from distinct samples or whether the same sample was measured repeatedly
☐	☒	The statistical test(s) used AND whether they are one- or two-sided<br>*Only common tests should be described solely by name; describe more complex techniques in the Methods section.*
☒	☐	A description of all covariates tested
☒	☐	A description of any assumptions or corrections, such as tests of normality and adjustment for multiple comparisons
☐	☒	A full description of the statistical parameters including central tendency (e.g. means) or other basic estimates (e.g. regression coefficient) AND variation (e.g. standard deviation) or associated estimates of uncertainty (e.g. confidence intervals)
☐	☒	For null hypothesis testing, the test statistic (e.g. *F*, *t*, *r*) with confidence intervals, effect sizes, degrees of freedom and *P* value noted<br>*Give P values as exact values whenever suitable.*
☒	☐	For Bayesian analysis, information on the choice of priors and Markov chain Monte Carlo settings
☒	☐	For hierarchical and complex designs, identification of the appropriate level for tests and full reporting of outcomes
☒	☐	Estimates of effect sizes (e.g. Cohen's *d*, Pearson's *r*), indicating how they were calculated

Our web collection on statistics for biologists contains articles on many of the points above.

Software and code

Policy information about availability of computer code

Data collection	No software or code was used in the data collection process.
Data analysis	All data analyses were conducted using MATLAB R2024a. All code for the global mesoscale front detection algorithm, along with our analysis methods for the two statistical indices used to quantify front-induced hotspot and barrier effects, is available at https://doi.org/10.5281/zenodo.17218778.

For manuscripts utilizing custom algorithms or software that are central to the research but not yet described in published literature, software must be made available to editors and reviewers. We strongly encourage code deposition in a community repository (e.g. GitHub). See the Nature Portfolio guidelines for submitting code & software for further information.

Data

Policy information about availability of data

All manuscripts must include a data availability statement. This statement should provide the following information, where applicable:

- Accession codes, unique identifiers, or web links for publicly available datasets
- A description of any restrictions on data availability
- For clinical datasets or third party data, please ensure that the statement adheres to our policy

Our findings are based on open-access datasets. Our global mesoscale front dataset, together with the identified frontal warm and cold zones, is available at https://zenodo.org/records/14785322. The global fishing effort dataset is available from https://globalfishingwatch.org. The satellite-observed SST dataset can be accessed at https://dx.doi.org/10.5285/4a9654136a7148e39b7feb56f8bb02d2. Argo and BGC-Argo profiling float data are available from https://doi.org/10.17882/42182.

The satellite-derived chlorophyll-a dataset can be accessed at https://doi.org/10.48670/moi-00281. All commercial fishing record data used in this study can be obtained by applying to the National Data Centre for Distant-water Fisheries of China or by requesting them from the corresponding authors, due to fishery data privacy. Source data are provided with this paper.

Research involving human participants, their data, or biological material

Policy information about studies with human participants or human data. See also policy information about sex, gender (identity/presentation), and sexual orientation and race, ethnicity and racism.

Reporting on sex and gender	Not applicable
Reporting on race, ethnicity, or other socially relevant groupings	Not applicable
Population characteristics	Not applicable
Recruitment	Not applicable
Ethics oversight	Not applicable

Note that full information on the approval of the study protocol must also be provided in the manuscript.

Field-specific reporting

Please select the one below that is the best fit for your research. If you are not sure, read the appropriate sections before making your selection.

- [ ] Life sciences
- [ ] Behavioural & social sciences
- [x] Ecological, evolutionary & environmental sciences

For a reference copy of the document with all sections, see nature.com/documents/nr-reporting-summary-flat.pdf

Ecological, evolutionary & environmental sciences study design

All studies must disclose on these points even when the disclosure is negative.

Study description	We applied a recently developed satellite-based front detection method that enables automated identification of both warm and cold zones of each front and established an analytical framework to quantify the frontal hotspot and barrier effects on fisheries. Using data on frontal zones, we classified each fishing location into frontal warm, cold, or whole frontal zones. These classifications were then used to quantify hotspot and barrier effects by comparing catch and fishing effort among these zones using two independent statistical indices: the relative anomaly difference of fishing catch (or effort) and the relative difference of fishing catch (or effort) per unit area. Additionally, based on actual fishery datasets, we generated 1,000 randomly distributed fishing datasets by simulating fishing locations within each local zone (2° boxes) to assess the significance of frontal hotspot and barrier effects on fisheries. Our results show that fisheries across diverse regions and major commercial stocks worldwide respond strongly to barrier effects, whereas responses to hotspot effects are generally sporadic. This suggests that frontal hotspot effects are less universal than previously assumed.
Research sample	We analyzed 109,186 daily commercial fishing records targeting chub mackerel, Japanese sardine, neon flying squid, and Pacific saury from Chinese fishing fleets during 2017–2021 in the northwest Pacific Ocean to quantify the frontal hotspot and barrier effects on fisheries. Fishery-independent cruise survey data collected between 2021 and 2024 from the Chinese research vessel Song Hang were used to support our findings. Additionally, we utilized satellite-based global industrial fishing data from Global Fishing Watch (GFW, https://globalfishingwatch.org) spanning 2012–2023 to examine frontal hotspot and barrier effects on fisheries across global regions and major commercial stocks.
Sampling strategy	Our fishery dataset including many commercial fishing records reaching commercial 109,186 records, which covering multiple years and mainly fishing grounds in the northwest Pacific Ocean. Global industrial fishing data are the biggest dataset with more than 1 billion fishing records. These datasets are sufficiently big to help us get the reliable findings. Our findings are consistently supported by three independent datasets analyzed using two independent statistical methods.
Data collection	Satellite-based global daily industrial fishing data were obtained from Global Fishing Watch (GFW, https://globalfishingwatch.org). This dataset is derived from convolutional neural networks trained on automatic identification system (AIS) data and provides daily information on fishing location, effort, gear type, and Maritime Mobile Service Identity (MMSI) for each vessel. Daily commercial fishing records from Chinese fishing fleets operating in the northwest Pacific Ocean were collected based on their electronic fishing logs. Fishery-independent cruise survey data were obtained from the Chinese research vessel Song Hang, operated by Shanghai Ocean University, using midwater trawling and squid jigging surveys. For a more detailed description, please refer to the Methods section of the manuscript.
Timing and spatial scale	Industrial fishing data from Global Fishing Watch provide daily fishing effort for all available fishing vessels across the global oceans, with a spatial resolution of 0.01° for the period 2012–2023. Chinese commercial fishing records include daily fishing locations and catches from 2017–2021, covering high-seas fishing grounds in the northwest Pacific Ocean. Fishery-independent cruise survey data provide species-specific catch-per-unit-effort (CPUE) measurements from 163 stations for midwater trawling and from additional stations for squid jigging, collected between June and July during 2021–2024.
Data exclusions	No data were excluded from the analyses.

Reproducibility	All findings can be reproduced using the open-access global industrial fishing dataset from Global Fishing Watch and our open-access global front dataset.
Randomization	Using data on frontal zones, we classified each fishing record into frontal warm, cold, or whole frontal zones based on its location. Our analysis did not involve any covariates.
Blinding	All fishing records objectively exist; therefore, our work did not involve blinding.

Did the study involve field work? ☐ Yes ☒ No

Reporting for specific materials, systems and methods

We require information from authors about some types of materials, experimental systems and methods used in many studies. Here, indicate whether each material, system or method listed is relevant to your study. If you are not sure if a list item applies to your research, read the appropriate section before selecting a response.

Materials & experimental systems

n/a	Involved in the study
☒	☐ Antibodies
☒	☐ Eukaryotic cell lines
☒	☐ Palaeontology and archaeology
☒	☐ Animals and other organisms
☒	☐ Clinical data
☒	☐ Dual use research of concern
☒	☐ Plants

Methods

n/a	Involved in the study
☒	☐ ChIP-seq
☒	☐ Flow cytometry
☒	☐ MRI-based neuroimaging

Plants

Seed stocks	Not applicable
Novel plant genotypes	Not applicable
Authentication	Not applicable
